# Immune reconstitution inflammatory syndrome as a cause of autoimmune hepatitis and acute liver failure

**DOI:** 10.5935/0103-507X.20170053

**Published:** 2017

**Authors:** Edison Moraes Rodrigues Filho, Rogério Fernandes, Ruth Susin, Bárbara Fior

**Affiliations:** 1 Liver Transplantation Group, Irmandade da Santa Casa de Misericórdia de Porto Alegre - Porto Alegre (RS), Brazil.; 2 Transplants Intensive Care Unit, Hospital Dom Vicente Scherer, Irmandade da Santa Casa de Misericórdia de Porto Alegre- Porto Alegre (RS), Brazil.; 3 Institutional Network of Research and Innovation on Intensive Medicine, Irmandade da Santa Casa de Misericórdia de Porto Alegre - Porto Alegre (RS), Brazil.; 4 Hospital de Clínicas de Porto Alegre - Porto Alegre (RS), Brazil.

**Keywords:** Immune reconstitution inflammatory syndrome, Hepatitis, autoimmune, Liver failure, acute, Case reports

## Abstract

Acute liver failure is a rare syndrome with high mortality and is often diagnosed late. Intensivist physicians play fundamental roles in the diagnostic suspicion and the management of the multiple-organic dysfunctions characteristic of this entity. Immune reconstitution inflammatory syndrome is an entity that is characterized by the paradoxical worsening of the patient's previous condition, after the initiation of antiretrovirals, triggered against either pathogens present in the host or autoantigens. Autoimmune hepatitis has recently been described as one of these autoimmune manifestations. The authors report the first case with evolution to acute liver failure and death within a few days after the development of encephalopathy, review the cases of autoimmune hepatitis described and comment on the therapeutic possibilities in this context.

## Abstract (pt)

A insuficiência hepática aguda é uma síndrome rara com elevada mortalidade e frequentemente reconhecida de forma tardia. Os médicos intensivistas desempenham um papel fundamental na suspeição diagnóstica e no manejo das disfunções múltiplo-orgânicas características desta entidade. A síndrome inflamatória de reconstituição imune é uma entidade que se caracteriza pela piora paradoxal do quadro prévio do paciente, após o início de antirretrovirais, desencadeada contra patógenos presentes no hospedeiro ou autoantígenos. A hepatite autoimune tem sido recentemente descrita como uma destas manifestações autoimunes. Os autores relatam o primeiro caso com evolução à insuficiência hepática aguda e óbito em poucos dias após o desenvolvimento de encefalopatia, revisam os casos de hepatite autoimune descritos e tecem comentários sobre as possibilidades terapêuticas neste contexto.

## INTRODUCTION

Acute liver failure is a rare syndrome with high mortality that is characterized by jaundice, encephalopathy occurring up to 26 weeks after detection of jaundice and coagulopathy (defined by a result of the international normalized ratio - INR - for prothrombin time ≥ 1.5) in the absence of previous liver disease.^(1)^ Despite the etiology, patients undergo massive necrosis and apoptosis of hepatocytes, systemic inflammatory response syndrome, and multiple organ dysfunction. Several different insults can trigger acute liver failure.^(2)^ Viral etiologies, including B-virus infection, are highlighted in Brazil. Management of patients with hepatic impairment depends on early recognition and involves etiological treatment, supportive treatment, and liver transplantation as appropriate to prognostic predictions.^(1)^

Immune reconstitution inflammatory syndrome (IRIS) is an entity that is characterized by the paradoxical worsening of the previous condition of a patient carrying the human immunodeficiency virus (HIV) after the initiation of antiretrovirals. It is an inflammatory response to pathogens present in the host, usually *Mycobacterium tuberculosis, Mycobacterium avium complex, Cryptococcus* sp., *Histoplasma capsulatum, Toxoplasma gondii, Pneumocystis jiroveci*, cytomegalovirus, herpesvirus, and John Cunningham virus (JC).^(3)^

Increased serum levels of transaminases may occur after the initiation of antiretrovirals in HIV-seropositive individuals with coinfection with B virus and/or C virus. However, a robust immune response may also be directed against autoantigens. This syndrome has been associated with several autoimmune conditions, suggesting that these conditions may be manifestations of immune reconstitution. These conditions include sarcoidosis, Graves' disease, systemic lupus erythematosus, rheumatoid arthritis, and thyroiditis, all occurring up to 1 year after the initiation of antiretrovirals. These individuals with autoimmune diseases secondary to immune reconstitution may be genetically predisposed.^(4)^

Autoimmune hepatitis is characterized by specific clinical and laboratory findings, such as elevated levels of autoantibodies, hypergammaglobulinemia, and microscopic liver changes, including interface hepatitis, plasmacytic infiltrate, and rosette-like regenerative liver cells.^(5)^ Autoimmune hepatitis is usually a chronic disease, but the clinical presentation may range from asymptomatic changes in liver function tests to acute liver failure.^(6)^

In the present article, we present the case of a patient with defined autoimmune hepatitis, attributable to immune reconstitution and with evolution to acute hepatic failure and death in a few days. We undertook a search for original articles reporting autoimmune hepatitis secondary to immune reconstitution after initiation of antiretrovirals. The search was performed in the PubMed and Embase databases without language and date restrictions. The keywords ((*Human immunodeficiency virus* OR HIV*-infected*) AND (*autoimmune hepatitis*)) were used, and 282 references were detected. After the exclusion of duplicate articles and the review of abstracts, four references were selected (Table 1). All of the patients in the selected reports were submitted to confirmatory biopsies of autoimmune hepatitis and were managed with corticosteroids and, eventually, azathioprine. All reported cases presented favorable responses to immunosuppression without progression to acute liver failure.

**Table 1:** Reports of autoimmune hepatitis due to immune reconstitution inflammatory syndrome in HIV-positive patients after antiretroviral initiation

Authors/year	N	Diagnostic time after ARTV initiation	Laboratory data	Treatment
O’Leary et al.^(7)^	1	8	ANA and SMA	Prednisone followed by azathioprine
Puius et al.^(8)^	2	7 and 3	IgG peak, ANA and SMA	Prednisone and azathioprine
Daas et al.^(9)^	1	6	IgG peak, ANA, anti-DNA and SMA	Corticotherapy
Murunga et al.^(10)^	9	3 to 18 (mean 5)	IgG Peak	Prednisone

ARTV - antiretrovirals; ANA - antinuclear antibodies; SMA - smooth muscle antibodies; IgG - immunoglobulin G.

## CASE REPORT

A 51-year-old woman with a history of HIV seropositivity identified in 2015, without previous AIDS-defining illnesses, and on antiretroviral treatment with tenofovir, 3TC and efavirenz since September 5, 2015, presented a baseline CD4 and viral load of 387 cells/mL and 268,000 copies/mL, respectively. Adequate antiretroviral response was documented due to elevation of CD4 levels to 417 cells/mL and a viral load drop to 94 copies/mL in January 2016. The patient had a history of epigastralgia, white feces, and coluria since the end of July 2016, with identification of jaundice on approximately August 4, 2016. Laboratory tests performed on August 10, 2016 showed the following changes: glutamic-oxalacetic transaminase (GOT) 1.863, glutamic-pyruvic transaminase (GPT) 895, total bilirubin (TB) 9.9, and direct bilirubin (DB) 7.6. Abdominal ultrasound was performed on August 12, 2016 and showed heterogeneous liver and gallbladder without stones. Hepatic function tests worsened on August 12, 2016, with a prothrombin time of 31% being documented, and hospital admission was indicated by an emergency department on the same date. On August 13, 2016, still in the emergency room, the patient developed grade III encephalopathy. Laboratory tests on August 14, 2016, demonstrated worsening of liver function tests, with an INR of 3.27. Viral serologies for B virus, A virus, and C virus, collected on August 10, 2016, were non-reactive. IgG antibodies to cytomegalovirus and herpesviruses were reactive but with non-reactive IgM antibodies. On August 15, 2016, the patient progressed to grade IV encephalopathy (Glasgow coma scale, 8; ocular opening, 2; verbal response, 1; and motor response, 5), requiring orotracheal intubation for airway protection; transfer to the intensive care unit was requested. Laboratory tests on August 16, 2016, showed TB of 13.8, INR of 5.23, and factor V of 22%. On the same day, the patient presented myoclonic seizures with secondary generalization. On August 16, 2016, the following results were made available: antinuclear factor (ANF) of 1:256 with a fine-dotted nuclear pattern, IgG of 2639 mg/dL, and anti-smooth muscle antibody-reactive of 1:80. Corticosteroids were started, and the patient was transferred to the intensive care unit. Computed tomography (CT) of the cranium was performed, which identified significant cerebral edema, progression to level 3 on the Glasgow coma scale, areflexia, and significant hemodynamic instability on the same day, which did not allow the achievement of the brain death protocol. The patient developed asystole and died on August 17, 2016. Hepatic biopsy *post mortem* identified massive hepatic necrosis and lymphoplasmacytic infiltrate.

## DISCUSSION

In our review, the 13 patients reported had acute hepatitis after the introduction of antiretrovirals, in the absence of viral and drug etiology justifying the condition.^(7-10)^ In these patients, there was laboratory and anatomopathological confirmation of autoimmune hepatitis similar to the case of our patient. The time relationship between antiretroviral initiation and the development of the clinical picture allows arguing in favor of IRIS as a triggering factor of the autoimmune hepatitis in this patient. In our case, no other autoimmune syndromes, such as vitiligo and thyroiditis, were identified to suggest a predisposition to the development of autoimmune hepatitis, although this predisposition cannot be ruled out *a priori*.

What differed between our patient's evolution and the other descriptions was the rapid development of acute liver failure in a few days, with hyperacute behavior and death likely secondary to refractory intracranial hypertension. Such an evolution did not allow either an earlier recognition of the severity of the condition at the emergency department or the institution of early immunosuppression with a favorable potential response, as described uniformly in the cases previously reported. Added to this catastrophic progression is the difficulty in performing an emergency transplantation in an HIV-positive patient, although several HIV-positive selected patients have had liver transplantation electively and with favorable evolution,^(11)^ including in our clinic. The non-use of HIV-positive donors for HIV-positive recipients in Brazil is another factor that limits the supply of organs to these patients in an emergency.

Therefore, considering the increasing prevalence of HIV-positive individuals on antiretroviral therapy in our country, it is fundamental to recognize changes in liver function tests early and to consider the possibility of autoimmune hepatitis in these patients once viral and drug etiologies are ruled out. In doubtful cases, without defined clinical-laboratory diagnosis, hepatic biopsy is indicated. Early recognition of autoimmune hepatitis may allow the administration of corticosteroids, with or without concomitant azathioprine, to avoid progression to chronic liver disease or even to acute liver failure, as in the case described here. Instead, the early recognition of signs of poor prognosis should lead to an indication of emergency liver transplantation, especially in individuals with a CD4 cell count > 100 cells/mL (or > 200 cells/mL if there is a previous history of opportunistic complications), an HIV viral load < 50 copies/mL (considering the ultrasensitive *Amplicor Monitor PCR* assay), absence of AIDS-defining diseases, absence of progressive multifocal leukoencephalopathy, absence of chronic intestinal cryptosporidiosis (duration greater than 1 month), or primary lymphoma of the central nervous system.^(11)^ A future review of the guidelines for the use of HIV-positive donors in our country that allows the use of these donors for HIV-positive recipients, as already accepted in other countries,^(12)^ may increase the opportunity for an emergency liver transplant in a timely manner in the presence of acute liver failure and poor prognostic criteria.

## CONCLUSION

In summary, the authors describe the case of an HIV-positive patient with acute liver failure due to autoimmune hepatitis, secondary to immune reconstitution inflammatory syndrome after the introduction of antiretroviral therapy. This is one of the few documented cases of this syndrome and the only one with evolution to acute liver failure. Future studies are needed to investigate the possibility that this syndrome is due to the acute presentation of undiagnosed autoimmune hepatitis prior to the introduction of antiretroviral therapy.
